# Steroid responsive encephalopathy associated with autoimmune thyroiditis (SREAT) presenting as major depression

**DOI:** 10.1186/s12888-016-0897-3

**Published:** 2016-06-06

**Authors:** Dominique Endres, Evgeniy Perlov, Oliver Stich, Ludger Tebartz van Elst

**Affiliations:** Section for Experimental Neuropsychiatry, Department of Psychiatry & Psychotherapy, Medical Center, University of Freiburg, Hauptstr. 5, 79104 Freiburg, Germany; Department of Neurology, Medical Center, University of Freiburg, Breisacher Str. 64, 79106 Freiburg, Germany

**Keywords:** SREAT, Depression, Thyroiditis, Corticosteroids

## Abstract

**Background:**

Hashimoto’s encephalopathy is a neuropsychiatric disease with symptoms of cognitive impairment, stroke-like episodes, seizures, and psychotic or affective symptoms associated with autoimmune thyroiditis and excellent steroid responsiveness; therefore, it is also called “steroid responsive encephalopathy associated with autoimmune thyroiditis” (SREAT).

**Case presentation:**

We present the case of a 50-year-old woman who developed a first-onset depressive syndrome with predominant cognitive impairment and inability to work. Antidepressive treatment and cognitive behavioral therapy over two years were unsuccessful. Neurological examination was unremarkable. Serum analysis showed increased thyroid peroxidase and thyroglobulin antibodies. Cerebrospinal fluid protein and albumin quotient were increased. Magnetic resonance imaging depicted unspecific, supratentorial white matter lesions and frontal accentuated brain atrophy. Electroencephalography was normal. Neuropsychological testing for attentional performance was below average. High-dose intravenous treatment with methylprednisolone over 5 days and oral dose reduction over 3 weeks led to the sustained improvement of clinical symptoms. Following discharge from the hospital, the patient returned to work, and 6.5 months after the start of therapy, no neuropsychological deficit remained.

**Conclusion:**

This case report illustrates that SREAT might present with purely depressive symptoms, thus mimicking classical major depression. In such cases, corticosteroid therapy may be an effective treatment option.

## Background

Hashimoto's encephalopathy is a neuropsychiatric disease with symptoms of cognitive impairment, stroke-like episodes including transient aphasia, tremor, myoclonus, gait disorders, or seizures [1]. It is associated with autoimmune thyroiditis and excellent steroid responsiveness and is therefore also called “steroid responsive encephalopathy associated with autoimmune thyroiditis” (SREAT). Usually, thyroid hormone abnormalities and, in particular, anti-thyroid peroxidase (TPO) and thyroglobulin (TG) antibodies are found. Here, we present the case of a patient with SREAT suffering from typical psychomotor and neurocognitive symptoms of a major depression, while neurological sequelae were absent. This case illustrates that SREAT without neurological deficits and with normal thyroid hormones can mimic major depression. It is important to be aware of this possible association between symptoms of the most common psychiatric disease (major depression) and the most frequent autoimmune disease (Hashimoto thyroiditis), because it implies specific and more causal treatment options, such as corticosteroids.

## Case presentation

We present the case of a 50-year-old female receptionist who, early in 2011, developed loss of energy and feelings of exhaustion but without any identified psychosocial stressors. At the end of the same year, she presented with a classical depressive syndrome (suffering from impaired concentration, slowed thinking processes, disturbed memory, low mood, decreased activity, reduced energy, fearfulness, symptoms of demoralization with hopelessness, reduced self-awareness, excessive guilty, sleep disturbance, inability to work, and social withdrawal). For the patient, the adynamia and cognitive impairment were the most debilitating symptoms. Hence, a major depression was diagnosed. Treatment with venlafaxine (up to 112.5 mg; higher doses were not tolerated) plus agomelatine (25 mg) together with cognitive-behavioral therapy was started without sufficient therapeutic success. In the autumn of 2012, occupational reintegration with reduced working hours was initiated, but it soon had to be stopped because of cognitive and also physical exhaustion. The patient would forget things and fail to understand more complex tasks. Reaction times were described as extended; memory was still disturbed, mood depressed, and energy level reduced.

In January 2014, the patient was admitted to our hospital. Her somatic history only showed high blood pressure, which was treated with telmisartan (80 mg), and a history of a clinically remitted lumbar disc prolapse. Thyroxine (T4) substitution had been started at the beginning of 2012 (because TPO and TG antibodies were elevated in an initial outpatient examination, whereas thyroid hormones were normal). On admission to our hospital, 75 μg T4 were taken. The patient had no history of psychiatric disorders prior to 2011, and her familial history was negative for psychiatric, neurological, or autoimmune disorders.

### Investigations

**Fig. 1:**
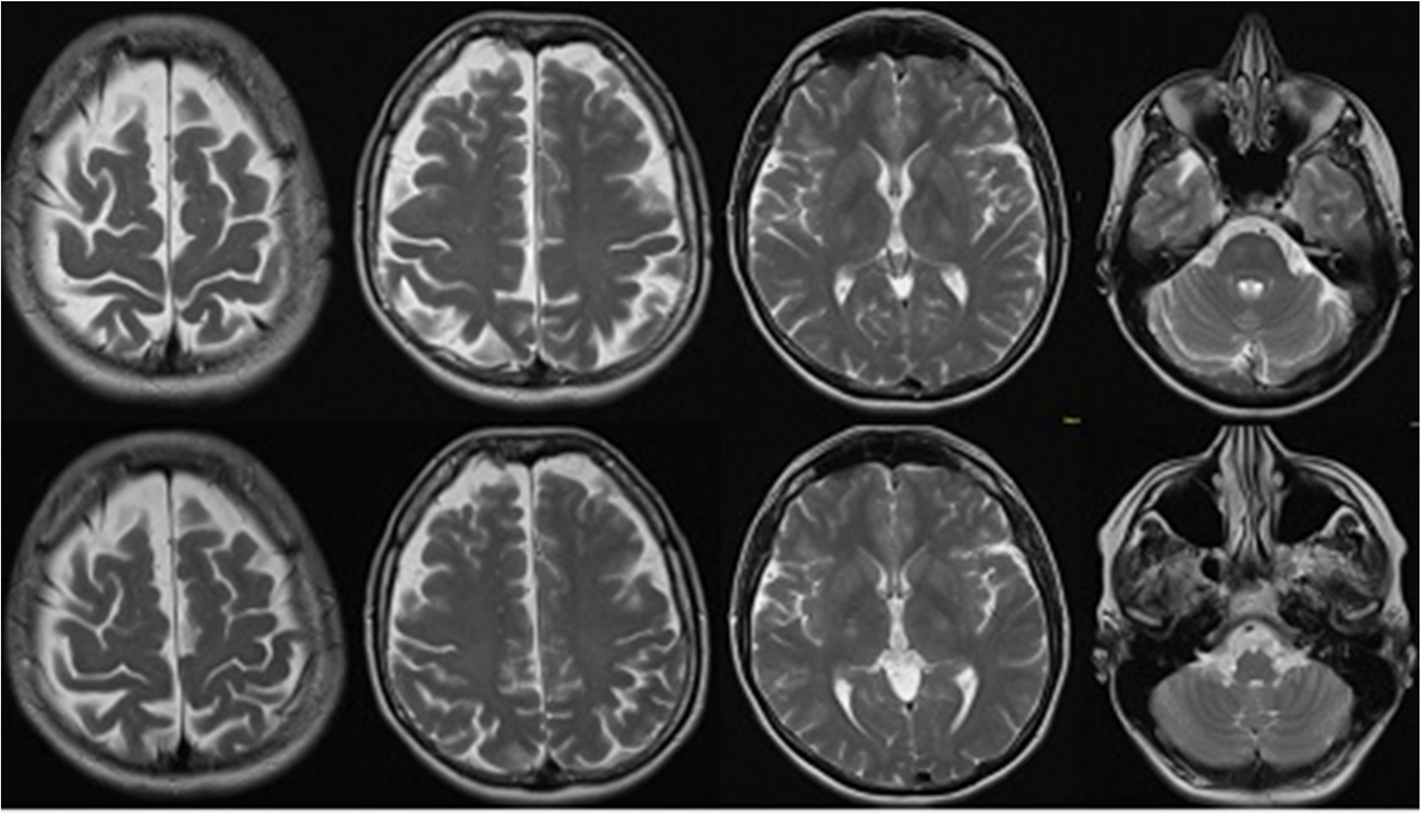
Magnetic resonance imaging (MRI; T2 weighted axial scans) before steroid treatment depicted unspecific supratentorial white matter lesions and frontal accentuated brain atrophy (upper row). Follow-up MRI 7 months after the start of therapy showed no changes (bottom row)

**Fig. 2:**
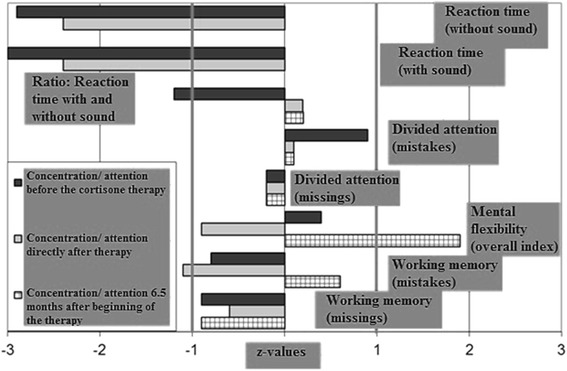
Tests for attentional performance measuring reaction time, divided attention, mental flexibility and working memory before (black bar), directly after (grey bar) and 6.5 months after cortisone therapy (shaded bar)

Internal and neurological examinations revealed no relevant abnormalities. Serum analysis showed increased TPO (804 IU/mL; normal range <34 IU/mL), increased TG (661.4 IU/mL; normal range <115 IU/mL), and normal thyroid-stimulating hormone (TSH) receptor antibodies. The levels of thyroid hormones (triiodothyronine [T3], T4 and TSH) were normal. No antibodies against intracellular onconeural antigens (Yo, Hu, CV2/CRMP5, Ri, Ma1/2, SOX1) or intracellular synaptic antigens (GAD, amphiphysin) were found. Cerebrospinal fluid (CSF) analysis illustrated a slight blood–brain barrier disturbance (protein 678 mg/L [reference <450 mg/L], albumin-quotient 7.7 [age-related reference < 7.3 × 10^−3^]), a normal white cell blood count (1/μL), and no intrathecal immunoglobulin synthesis or oligoclonal bands. Moreover, there were no antibodies against neuronal cell surface antigens (NMDAR, AMPA-R, GABA-B-R, VGKC-complex [LGI1, Caspr2]) in the CSF. Magnetic resonance imaging (MRI) depicted unspecific supratentorial white matter lesions and frontal accentuated brain atrophy (Fig. 1). Electroencephalography (EEG) was normal. In cognitive testing for attentional performance (TAP), response time was greatly reduced and alertness and processing speed were below average (Fig. 2).

### Differential diagnosis

The most important differential diagnosis was major depression, because the main symptoms of this were present (lowering of mood, reduction of energy, and decrease in activity [www.dsm5.org]). The leading symptoms were cognitive impairments, including forgetfulness (in particular for short-term memory), so pre-senile dementia also had to be considered. Other reasons for inflammatory brain diseases, such as limbic encephalitis or metabolic disorders, were excluded [2].

### Treatment

Intravenous treatment with high dose methylprednisolone 1000 mg over 3 days and 500 mg over 2 days was provided and well tolerated. Methylprednisolone treatment was continued with 40 mg orally and tapered by halving the daily dose every fifth day. Treatment with venlafaxine (112.5 mg), agomelatine (25 mg), and T4 (75 μg) was continued unchanged.

### Outcome and follow-up

Directly after the high-dose intravenous steroid treatment, the patient reported reduced cognitive impairment and improved alertness. Neuropsychological testing confirmed this rapid improvement with reduced response latencies in all attention tasks, compared with measurement before corticosteroid treatment. The basal alertness and processing speed was improved, but still below-average. After around five weeks, however, the mood and energy levels had normalized and cognitive impairment disappeared. After three months, the patient was fully reintegrated at work without cognitive deficits. In the follow-up testing for attentional performance (6.5 months after therapy onset), no relevant neuropsychological deficit remained (Fig. 2). No changes in the follow-up MRI were detected (Fig. 1). TPO (440.6 IU/mL; initial 804 IU/mL) and TG antibodies (581.7 IU/mL; initial 661.4 IU/mL) decreased but still exceeded the reference limit.

## Conclusions

SREAT was first described in 1966 [3], and several case reports and series have been described since [1, 4, 5]. To date, no definite diagnostic criteria have been established for SREAT [6]. Preliminary suggestions remain very vague with respect to the question of which precise psychiatric symptoms and syndromes should lead to the diagnoses when thyroid antibodies are present [7]. Typical SREAT symptoms include not only neuropsychiatric syndromes with behavioral or cognitive abnormalities (in nearly all cases, and including ours), but also tremor (80 %), stroke-like episodes including transient aphasia (80 %), myoclonus (65 %), gait ataxia (65 %), seizures (60 %), and sleep abnormalities (55 %) [1]. Only a few case reports have documented predominant depressive symptoms [8–12]. In an overview of five such cases until 2013, only one presented with isolated affective symptoms [8]. A case report from our clinic described a patient with predominant depressive symptoms as well as an epileptic seizure [9]. Laske et al. described a 74-year-old female patient with depression and EEG slowing; after steroid treatment, the affective symptoms normalized parallel with the EEG [10]. Other psychiatric cases have been associated with neurological signs, such as myoclonic jerks or ataxia [11]. SREAT can also be found in children, often associated with epilepsy, but in single cases also, with primarily behavioral presentations [12].

Here we present a unique SREAT case, one that presented clinically only with typical symptoms of a major depression. Remarkably, the levels of T3, T4, and TSH were normal, as in some earlier reports [1, 2]. Thus, as pointed out by Castillo et al. it is very unlikely that T3, T4, and TSH are directly involved in the pathophysiology of SREAT. By definition of SREAT, TPO and also TG antibodies are elevated; however, it is known that these levels do not correlate with the relevant symptoms [1]. Antibodies are not considered to be directly involved in the pathophysiology of SREAT, because they can also be found elevated in healthy subjects or in patients with other autoimmune diseases [2]; also, in line with this, antibodies were still elevated after remission of depressive symptoms in our patient. The CSF protein level and albumin quotient in our subject were increased, as generally described in earlier reports [1]. EEG findings were normal in our subject. This contrasts to the about 95 % of SREAT patients with pathological EEGs in earlier case summaries with generalized slowing, indicating diffuse brain dysfunction, being the most common finding [1]. The normal EEG along with the absence of other neurological deficits in our case might be a marker of a less severe variant of SREAT or, alternatively, the consequence of an early initial diagnosis. The MRI indicated unspecific changes, as noted in the majority of published cases [1]. In our patient, the presence of TPO and TG antibodies in combination with an excellent response to steroid treatment helped to establish the correct diagnosis after careful exclusion of other relevant disorders.

In our view, therefore, this case and literature review illustrates that TPO and TG antibodies should be performed routinely in depressive patients with atypical symptoms (discrete neurological symptoms like tremor or myoclonic jerks) and predominant cognitive impairments, because SREAT may manifest with just symptoms of major depression plus increased thyroid antibodies. Moreover, CSF analysis, EEG, and MRI might be helpful in establishing the correct diagnosis. This is particularly important due to a high association between TPO antibody levels and depressive episodes [13]. In a queried case of SREAT, a probatory therapy course with steroids is the only way to clarify the diagnosis.

## Abbreviations

CSF, cerebrospinal fluid; EEG, electroencephalography; MRI, magnetic resonance imaging; SREAT, steroid responsive encephalopathy associated with autoimmune thyroiditis; T3, triiodothyronine; T4, thyroxine; TAP, testing for attentional performance; TG, thyroglobulin; TPO, thyroid peroxidase; TSH, thyroid-stimulating hormone
